# Validation of an emotional model by EEG recordings of neural responses

**DOI:** 10.1186/1471-2202-14-S1-P379

**Published:** 2013-07-08

**Authors:** Nicoladie D Tam

**Affiliations:** 1 Department of Biological Sciences, University of North Texas, Denton, TX 76203, USA

## 

This study provides an experimental confirmation of the proportionality hypothesis predicted by the theoretical model of emotion [1,2] based on EEG recordings. The computational emotional model [1,2] proposed that emotion is an error-feedback signal provided to an organism to estimate the accuracy of the internal brain prediction of external world. It is based on the evolutionary principle of survival in which the survivability of an organism increases with the accuracy of the prediction of the external events. For an autonomous organism, any inaccuracy of the internal brain model (and/or inaccuracy of the perceptual input or execution output) needs to be self-discovered and self-corrected to function independently. This process of self-discovered errors is facilitated by self-directed error-signals - by comparing the predicted reality with the actual reality. The greater the disparity, the greater the potential internal error could be. Thus, the hypothesis posed by this emotional model is that emotional intensity is proportional to the perceived disparity between the expected reality and the actual reality. The size of emotional intensity signal signifies to the organism the severity of the error conditions that need to be self-corrected later in order to increase its survivability. Emotional feedback is a process in which such self-correction of faults within the system can be accomplished unassisted (for any autonomous organism).

The proportionality relationship between emotional intensity and perceived disparity in sharing in a social interaction has been confirmed by using the ultimatum game paradigm (UG) [3] based on self-reported psychological measures. To further validate the emotional model by recording neural signals from the brain, the present study used 14-channel wireless EEG recordings to detect the differences in neural responses with respect to the perceived disparities. The UG paradigm [4] is used to elicit an emotional response to a quantifiable disparity between the expected and the actual outcomes in sharing (splitting an amount of money) in social interaction. The intensity of anger the self-reported response is directly proportional to the amount of disparity (unfairness) in the monetary offer-ratio between the proposer and the responder in the UG game, as previously reported [3]. Such proportionality relationship also changes dependent on whether the offer is favorable (hyper-fair) or unfavorable (unfair) to the responder [3]. The computational model quantifies how emotional sensitivity is changed accordingly, which is represented by the change in the slope of the emotion-disparity function. That is, subjects are more sensitive to anger when the offer is unfavorable (unfair) to them than favorable (hyper-fair) offer. This is consistent with the prediction of the theoretical model that greater emphasis is placed on error-conditions that are unfavorable to self (enhancing selective attention to correct errors that are self-disadvantage) than error-conditions that are favorable (advantageous) to self.

The results confirmed similar finding of proportionality relationship between the amplitude of the evoked EEG signals and the disparity in offer-ratio. This provided independent confirmation of the emotional model using electrophysiological measurements to detect the neural response to the disparity signal in social sharing. This provides further evidence supporting the proportionality hypothesis using electrophysiological measures of neural responses, in addition to the previous psychological study using self-report rating as a measure.

## References

[B1] Tam D EMOTION-I Model: A Biologically-Based Theoretical Framework for Deriving Emotional Context of Sensation in Autonomous Control Systems The Open Cybernetics and Systemics Journal 2007 1 28 46 10.2174/1874110X00701010028

[B2] Tam D EMOTION-II Model: A Theoretical Framework for Happy Emotion as a Self-Assessment Measure Indicating the Degree-of-Fit (Congruency) between the Expectancy in Subjective and Objective Realities in Autonomous Control Systems The Open Cybernetics and Systemics Journal 2007 1 47 60 10.2174/1874110X00701010047

[B3] Tam DN Computation in Emotional Processing: Quantitative Confirmation of Proportionality Hypothesis for Angry Unhappy Emotional Intensity to Perceived Loss Cognitive Computation 2011 3 2 394 415 10.1007/s12559-011-9095-2

[B4] Von Neumann J Morgenstern O Theory of games and economic behavior 1953 Princeton: Princeton University Press

